# Voluntary Movement Frequencies in Submaximal One- and Two-Legged Knee Extension Exercise and Pedaling

**DOI:** 10.3389/fnhum.2016.00036

**Published:** 2016-02-05

**Authors:** Julie Stang, Håvard Wiig, Marte Hermansen, Ernst Albin Hansen

**Affiliations:** ^1^Department of Sports Medicine, Norwegian School of Sport SciencesOslo, Norway; ^2^Department of Physical Performance, Norwegian School of Sport SciencesOslo, Norway; ^3^Research Interest Group of Physical Activity and Human Performance, SMI^®^, Department of Health Science and Technology, Aalborg UniversityAalborg, Denmark

**Keywords:** cycling cadence, motor control, preferred pedaling rate, rhythmic movement, voluntary motor behavior

## Abstract

Understanding of behavior and control of human voluntary rhythmic stereotyped leg movements is useful in work to improve performance, function, and rehabilitation of exercising, healthy, and injured humans. The present study aimed at adding to the existing understanding within this field. To pursue the aim, correlations between freely chosen movement frequencies in relatively simple, single-joint, one- and two-legged knee extension exercise were investigated. The same was done for more complex, multiple-joint, one- and two-legged pedaling. These particular activities were chosen because they could be considered related to some extent, as they shared a key aspect of knee extension, and because they at the same time were different. The activities were performed at submaximal intensities, by healthy individuals (*n* = 16, thereof eight women; 23.4 ± 2.7 years; 1.70 ± 0.11 m; 68.6 ± 11.2 kg). High and fair correlations (*R*-values of 0.99 and 0.75) occurred between frequencies generated with the dominant leg and the nondominant leg during knee extension exercise and pedaling, respectively. Fair to high correlations (*R*-values between 0.71 and 0.95) occurred between frequencies performed with each of the two legs in an activity, and the two-legged frequency performed in the same type of activity. In general, the correlations were higher for knee extension exercise than for pedaling. Correlations between knee extension and pedaling frequencies were of modest occurrence. The correlations between movement frequencies generated separately by each of the legs might be interpreted to support the following working hypothesis, which was based on existing literature. It is likely that involved central pattern generators (CPGs) of the two legs share a common frequency generator or that separate frequency generators of each leg are attuned via interneuronal connections. Further, activity type appeared to be relevant. Thus, the apparent common rhythmogenesis for the two legs appeared to be stronger for the relatively simple single-joint activity of knee extension exercise as compared to the more complex multi-joint activity of pedaling. Finally, it appeared that the shared aspect of knee extension in the related types of activities of knee extension exercise and pedaling was insufficient to cause obvious correlations between generated movement frequencies in the two types of activities.

## Introduction

Better understanding of behavior and control of common voluntary human rhythmic movements is useful in the work to improve performance, function, and rehabilitation of exercising, healthy, and injured humans. Examples of human voluntary stereotyped rhythmic leg movements such as walking (Shapkova, 2004; Minassian et al., 2007), running (Cappellini et al., 2006; Sardroodian et al., 2015), and pedaling (Zehr et al., 2007; Hansen et al., 2014) are widely considered to be controlled by spinal neural networks, termed central pattern generators (CPGs). These CPGs are assisted by tonic supraspinal input and afferent feedback (for review, see previous publications Zehr and Duysens, 2004; Prochazka and Ellaway, 2012). In brief, the internal organization of a CPG is considered to be functionally separated into two components. One of the components is responsible for generation of a rhythmic movement frequency while another is responsible for a rhythmic movement pattern (Perret and Cabelguen, 1980; Kriellaars et al., 1994; McCrea and Rybak, 2008; Dominici et al., 2011). It is further considered that there are separate CPGs for each of the two legs (Shapkova, 2004; Zehr, 2005). Though, many details remain unknown (Ivanenko et al., 2006; Daun-Gruhn, 2011; Hansen, 2015). For example, our knowledge is limited on how common the CPG-generated rhythmogenesis is for man’s two legs when considering one- and two-legged systems. Studies of CPG-generated voluntary rhythmic movement in healthy humans are obviously challenged by researchers’ limited access to the spinal cord, as compared with studies on animals. Animal studies enable both stimulation of CPGs, measurement of CPG activity, and measurement of behavior. Though, in order to be able to further develop these considerations, analyses of human rhythmic movement behavior may be applied (Goulding, 2009; Schlinger, 2015), which is what was done in the present study.

The overall aim of the present study was to add to the understanding of movement behavior and control in human voluntary stereotyped rhythmic leg movements. In order to meet this objective, correlations between freely chosen movement frequencies in relatively simple, single-joint, one- and two-legged knee extension exercise were investigated. The same was done for more complex, multiple-joint, one- and two-legged pedaling. In case that the present study results in high correlations between movement frequencies generated separately by each of the legs it might be interpreted to support a working hypothesis of the two legs’ CPGs to share a common frequency generator, or alternatively, of separate frequency generators of each of the legs to be attuned via interneurons. The present involvement of two related, yet different, types of activities of knee extension exercise and pedaling also allowed the investigation of the following two test hypotheses: (i) The degree of common frequency generation for the two legs (i.e., the correlation coefficients of correlations between frequencies for the legs) is similar in the activity of knee extension exercise and the activity of pedaling. (ii) The shared aspect of knee extension, in itself, in the related types of activities of knee extension exercise and pedaling is sufficient for the generated movement frequencies in the two types of activities to correlate at least moderately. Knee extension contributes to characterize the down stroke phase in pedaling where most of the external power is produced (So et al., 2005).

## Materials and Methods

### Participants

Sixteen healthy individuals (8 women, 8 men; 23.4 ± 2.7 years; 1.70 ± 0.11 m; 68.6 ± 11.2 kg) volunteered to participate in the study. They were carefully informed about the procedures of the study and the overall aim (“to enlarge our knowledge on control of rhythmic movement”) but at the same time kept naive to the specific purpose. The latter served to avoid any particular conscious movement control during the applied activities. All participants occasionally performed cycling as personal transport or exercise, but none of them were competitive cyclists. Fourteen of the participants reported that the right leg was their dominant leg while two participants reported that the left leg was their dominant leg. Written informed consent was obtained from all participants. The present study was carried out in accordance with the recommendations of the regional committee of Southern Norway for research ethics and the study conformed to the standards set by the Declaration of Helsinki.

### Test Session

Each participant was informed to abstain from alcohol and tobacco 24 h before the test session. In addition, the participant was informed not to perform any intensive exercise 48 h before the test session.

Each participant reported to the laboratory for a single test session that lasted 1 h and consisted of the following. First, the participant was allowed to adjust the cycle ergometer with respect to seat and handlebar position. Second, warm up and familiarization to the knee extension activities were performed in form of 5 min of two-legged knee extension exercise followed by 3 min of rest and 5 min of one-legged knee extension exercise. After another 3 min of rest, six activities were performed in random order as 5 min-bouts interrupted by 3 min-rest periods. The six activities consisted of one- and two-legged knee extension exercise as well as one- and two-legged pedaling. The two-legged knee extension exercise was performed in a way that each of the legs was extended in an alternating way as in pedaling. Each participant was informed in “not thinking of anything special” and “letting the legs move by themselves” during the activities. Frequency was freely chosen in all activities. During the activities, the participant was blinded to the frequency and the time.

For knee extension exercise, a custom build electromagnetically braked ergometer was applied (Hallén et al., 1996). In brief, the ergometer consisted of a car seat placed above an electromagnetically braked cycle ergometer, which had aluminum rods mounted on the crank arms instead of pedals. The rods pointed forwards with respect to the seated participant. Braces were attached to the other ends of the rods. The participants’ feet were placed in the braces. The knee extension ergometer was constructed in a way that the flywheel only could be pulled, by knee extension, and not pushed, by knee flexion. Further, the power output that was delivered to the ergometer was a linear function of flywheel speed. For the present study, the ergometer was programmed for a power output (in W) during one-legged knee extension that corresponded to body mass times 0.5 and 0.6 for women and men, respectively, at a frequency of 60 rpm. Power output during two-legged knee extension exercise was twice as high. The construction of the ergometer resulted in power output to be higher or lower if the participants chose a higher or lower, respectively, frequency than 60 rpm during knee extension exercise. The participant’s torso was strapped to the seat during knee extension. For pedaling, an electromagnetically braked Excalibur Lode cycle ergometer (Lode BV, Groningen, Netherlands) was applied. The participant wore his or her own sports shoes and the pedals were mounted with toe-clips. Power output during one-legged pedaling corresponded to body mass times 1.0. Power output during two-legged pedaling was twice as high. It applies to both knee extension exercise and pedaling that frequency was calculated as an average across the last 2 min of a bout.

Heart rate was measured by a Polar F6 (Electro Oy, Kempele, Finland) every 30th s during each bout and an average value was subsequently calculated across each bout. Rate of perceived exertion (RPE) was indicated by the participant immediately after the bout. For this, a 15-grade Borg RPE scale with scores from 6–20 and corresponding subjective ratings from “Very light” (9 on the scale) to “Maximal exertion” (20 on the scale) was applied (Borg, 1970).

The specific outcome measures of the present study were pedaling and knee extension frequencies, power output, heart rate, and RPE.

### Statistics

**Figure 1:**
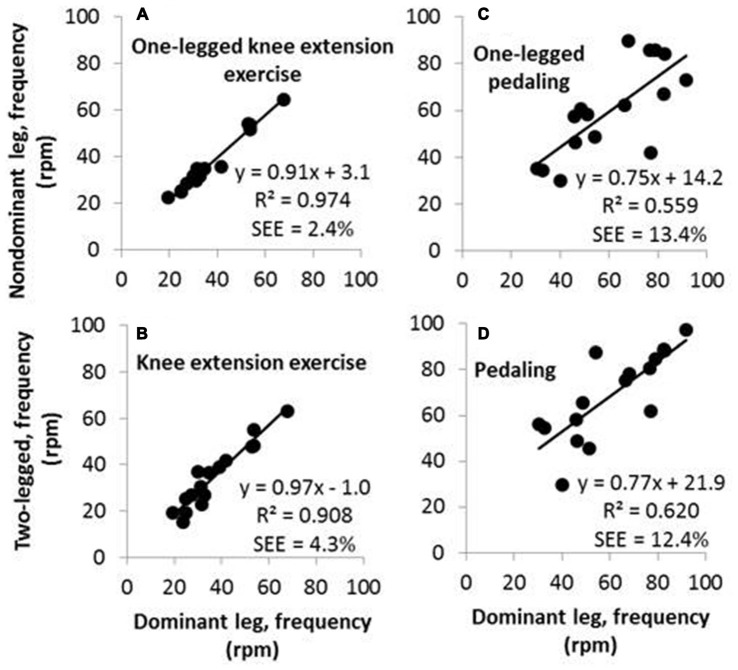
**Upper panels contain scatterplots of movement frequencies of the nondominant leg as a function of movement frequencies of the dominant leg in the relatively simple single-joint activity of one-legged knee extension exercise (A) and in the more complex multi-joint activity of one-legged pedaling (C).** Lower panels contain scatterplots of movement frequencies of two legs as a function of frequencies of the dominant leg in knee extension exercise **(B)** and in pedaling **(D)**.

Pearson product-moment correlation coefficients (*R*-values) were applied for investigation of the correlation between movement frequencies. Linear regressions were performed for associations presented in Figure 1. Paired-samples *t*-tests were applied for evaluations of potential differences in average values. The ratings based on the size of the *R*-values apply to both positive and negative correlations as follows: ≤0.25 is considered weak, 0.26–0.50 is moderate, 0.51–0.75 is fair, and ≥0.76 is high (Berg and Latin, 2008). The *R*-values were tested for significance, i.e., the probability of obtaining the correlation coefficient by chance.^1^ Statistics were calculated in Excel 2010 (Microsoft Corporation, WA, USA). Data are presented as average ± SD, unless otherwise indicated. Statistical significance was set at *p* < 0.05.

## Results

### Power Output

During the one- and two-legged knee extension exercise, power output was 19 ± 5 and 37 ± 9 W, respectively. During one- and two-legged cycling, it amounted to 68 ± 11 and 137 ± 22 W, respectively.

### Heart Rate and RPE

Heart rate values were in general low to moderate, which confirmed the submaximal character of the performed activities (Table 1). By estimating maximal heart rate as 208 – 0.7 × age (Tanaka et al., 2001), average relative heart rates amounted to 68% of maximum, or less. In line with that, RPE values were in general low to moderate. Thus, the highest average score amounted to 12.6, which indicated perceived exertion between “Fairly light” and “Somewhat hard”, based on the applied Borg RPE scale (Table 1).

**Table 1:** **Heart rate and RPE presented as average ± SD**.

Activity	Heart rate (beats per min)	RPE (Borg scale value)
Knee extension exercise, one-legged (ND)	86 ± 25	8.6 ± 2.8
Knee extension exercise, one-legged (D)	86 ± 23	8.9 ± 2.1
Knee extension exercise, two-legged	89 ± 26	8.8 ± 2.2
Pedaling, one-legged (ND)	120 ± 15	12.3 ± 2.2
Pedaling, one-legged (D)	122 ± 17	12.4 ± 2.3
Pedaling, two-legged	138 ± 16*	12.6 ± 2.4

ND, nondominant; D, dominant. *Different from both one-legged pedaling activities (p = 0.00001 and 0.0003).

### Movement Frequencies

Freely chosen knee extension frequencies ranged from 15–68 rpm across all participants and all three knee extension exercise activities. There was a high correlation between frequencies generated with the dominant leg and the nondominant leg (Figure 1A and Table 2). Further, there were high correlations between frequencies performed with each of the two legs, separately, and the frequency performed in two-legged knee extension exercise (Figure 1B and Table 2).

**Table 2:** **Heart rate and RPE presented as average ± SD**.

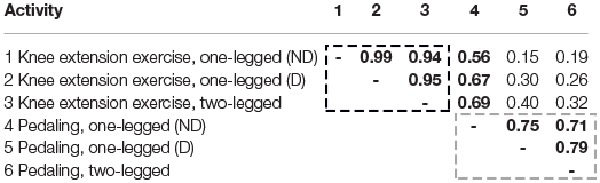

For comparison, freely chosen pedaling frequencies ranged from 30–97 rpm across all participants and all three pedaling activities. There was a fair correlation between pedaling frequencies performed with the dominant leg and the nondominant leg (Figure 1C and Table 2). Further, there were fair to high correlations between pedaling frequencies performed with each of the two legs, separately, and the frequency performed in two-legged pedaling (Figure 1D and Table 2). It applies to all *R*-values from correlations between pedaling frequencies (0.71–0.79) that they were lower than the *R*-values from correlations between knee extension frequencies (0.94–0.99; Table 2).

Pedaling frequencies performed in one-legged pedaling with the nondominant leg showed fair correlations (*R*-values between 0.56 and 0.69) with movement frequencies in all knee extension exercise activities (Table 2). But otherwise, there were no significant correlations between pedaling frequencies and knee extension frequencies.

## Discussion

The present study supports the previous finding that freely chosen movement frequency is considerably individual in voluntary stereotyped rhythmic leg movement such as in pedaling (Sarre et al., 2003). In addition to the individual character, such a freely chosen movement frequency appears to be consistent within individuals. As an example, it has been reported that freely chosen pedaling frequency is highly reliable across bouts, within a test session (Hansen et al., 2002), as well as across days (Hansen and Ohnstad, 2008). Of particular note is that the freely chosen knee extension frequency in the present study ranged considerably. Thus, the participant with the highest knee extension frequency worked at an around 350% higher frequency than the participant with the lowest frequency. That is considerably more than what has been observed for other stereotyped rhythmic movements of e.g., finger tapping (approx. 205%) and two-legged pedaling (approx. 183%; Hansen and Ohnstad, 2008). The reason is unknown and must be investigated in future studies, but the finding is not considered to affect the overall interpretation of the present results.

**Figure 2:**
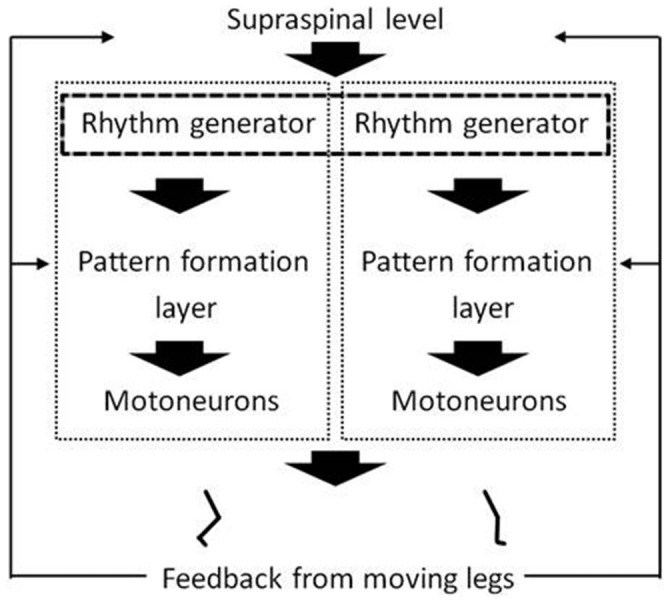
**A hypothetical multi-layered organization of rhythm and pattern generators in the spinal cord under influence of descending and sensory input.** The figure includes central pattern generators (CPGs) of both legs. The dashed rectangle that frames the rhythm generators is supposed to illustrate the hypothesis that the two legs’ CPGs share a common frequency generator, or alternatively, that the separate frequency generators are attuned via interneurons. The gray to black color of the dashed rectangle illustrates the hypothesis that the degree of *how* common the frequency generation is, is task- or activity-dependent. The figure is inspired by previous work (McCrea and Rybak, 2008; Lacquaniti et al., 2012, 2013).

The present fair to high correlations between movement frequencies generated separately by each of the legs during pedaling and knee extension exercise, respectively, could be interpreted as support of the working hypothesis that the two legs’ CPGs share a common frequency generator. Or alternatively, that separate frequency generators of each of the legs are attuned via interneurons. That hypothesis is illustrated in Figure 2. Existence of separate CPGs for each leg, and the possibility of activating them separately or in combination, has been suggested previously based on studies of leg movements in completely and incompletely paralyzed patients (Shapkova, 2004). For example, Shapkova (2004) reported unilateral stepping-like movements with spinal cord electrical stimulation of the lumbar enlargement. The present findings obtained on healthy subjects support and add to Shapkova’s findings on patients. In addition they supplement research focusing on CPG- organization and -function in lower animals.

A considerable common rhythmogenesis for the two legs could further be suggested based on the present fair to high correlations between frequencies performed in a certain activity with each of the two legs, separately, and the frequency performed in two-legged activity in the same activity.

The activity of pedaling is to a degree characterized by knee extension. Thus, knee extension contributes to characterize the down stroke phase in pedaling where most of the external power is produced. Based on that, it could be hypothesized that generated movement frequencies in pedaling and knee extension exercise might strongly correlate. However, the present study indicated that this did not appear to be the case. Rather, the type of task, or activity, appeared to be of relevance in the present study. Firstly, the common between-leg rhythmogenesis appeared to be strong for knee extension exercise, which may be characterized as relatively simple, single-joint, exercise. For comparison, the common between-leg rhythmogenesis appeared to be weaker for pedaling, which may be characterized as a more complex activity involving multiple joints. An interpretation of this might be that the two legs’ CPGs shared a common rhythmogenesis, however, that this characteristic became attenuated during leg movement that involved more segments, joints, and muscles, i.e., in pedaling. Reasons for that could involve differences in degrees of complexity, afferent feedback, and supraspinal input. Another reason that type of activity appeared to be of relevance is the following. There appeared to be only minor sharing of between-activity rhythmogenesis when comparing knee extension exercise and pedaling. For comparison, it has recently been reported that freely chosen stride frequencies in related activities of walking and running correlated (*R* = 0.72, *p* < 0.001) in a group of 27 recreationally active individuals. However, that at the same time, freely chosen pedaling frequency was not correlated with stride frequency in each of the locomotion types of walking and running (Sardroodian et al., 2015). A joint interpretation of those previous, and the present, results could be that when two activities at a point become adequately different, the sharing of between-activity rhythmogenesis attenuates.

The frequency during one-legged pedaling with the nondominant leg showed fair correlation with frequencies during all three types of knee extension exercise. Interestingly, the same was not the case for one-legged pedaling with the dominant leg. This difference is difficult to explain. But, perhaps it indicates that rhythmogenesis during one legged pedaling with the nondominant leg, in particular, to a degree is “driven” by CPGs responsible for rhythmogenesis during the simpler knee extension exercise.

The present type of investigation has some built-in interpretational challenges. For example, it is not possible to directly measure a gross frequency output from a human CPG with available techniques. Here, we suggest that the observed movement frequencies are net, or resultant, outputs from CPGs, i.e., accounting for influence from in particular afferent feedback and supraspinal input. Regarding the complexity of the applied types of activities, it should be noted that during pedaling activities, there is both a pushing phase in which the foot is pushed forward at the top dead center, a down stroke phase where most of the external power is produced, and an upstroke phase in which the leg is partly lifted (So et al., 2005). The down stroke phase is characterized by extension of the knee. Knee extension exercise is simpler in the way that the power production phase and the upstroke phase are coinciding and performed with the same muscles. Less segments, muscles, and joints are involved in knee extension exercise as compared to pedaling that should qualify the former to be characterized as simpler (Daun-Gruhn, 2011). For the present study, participants adjusted the cycle ergometer settings themselves. It cannot be ruled out that results would have been altered had e.g., a certain relative seat height been applied. Still, most importantly, seat height was maintained across pedaling conditions for each participant.

In conclusion, fair to high correlations occurred between freely chosen movement frequencies generated separately by each of the legs. This might be interpreted to support the working hypothesis that the involved CPGs of the two legs share a common frequency generator or that separate frequency generators of each of the legs are attuned via interneuronal connections. Further, the apparent common rhythmogenesis for the two legs appeared to be stronger for the relatively simple single-joint activity of knee extension exercise as compared to the more complex multi-joint activity of pedaling. Finally, it appeared that the shared aspect of knee extension in the related activity types of knee extension exercise and pedaling was insufficient to cause obvious correlations between generated movement frequencies in the two types of activities.

## Author Contributions

EAH had the initial idea for the study. All authors contributed to the planning of the study design. JS, MH and HW performed the data collection. All authors contributed on the data analyses and the intepretation of the results. EAH completed the first draft of the article. All authors have participated in revising of draft to finalize the manuscript.

## Conflict of Interest Statement

The authors declare that the research was conducted in the absence of any commercial or financial relationships that could be construed as a potential conflict of interest. The Review Editor Dr. Pedro Figueiredo declares that, despite being affiliated with the same institution as the Associate Editor Dr. Jae Kun Shim, the review process was handled objectively.

## Footnotes

^1^http://vassarstats.net/tabs.html
